# A Correlation Study of DHA Dietary Intake and Plasma, Erythrocyte and Breast Milk DHA Concentrations in Lactating Women from Coastland, Lakeland, and Inland Areas of China

**DOI:** 10.3390/nu8050312

**Published:** 2016-05-20

**Authors:** Meng-Jiao Liu, Hong-Tian Li, Li-Xia Yu, Gao-Sheng Xu, Hua Ge, Lin-Lin Wang, Ya-Li Zhang, Yu-Bo Zhou, You Li, Man-Xi Bai, Jian-Meng Liu

**Affiliations:** 1 Institute of Reproductive and Child Health/Ministry of Health Key Laboratory of Reproductive Health, Peking University Health Science Center, 38 Xueyuan Rd., Beijing 100191, China; liumengjiao_bjmu@163.com (M.-J.L.); liht@bjmu.edu.cn (H.-T.L.); linlinwang@bjmu.edu.cn (L.-L.W.); zhangyl@bjmu.edu.cn (Y.-L.Z.); zhouyubo@yeah.net (Y.-B.Z.); liyou@pku.edu.cn (Y.L.); 2 Department of Epidemiology and Biostatistics, School of Public Health, Peking University Health Science Center, 38 Xueyuan Rd., Beijing 100191, China; 3 Department of Obstetrics and Gynaecology, Weihai Maternal and Child Health Hospital, 51 Guangming Rd, Weihai 264200, China; Whyulixia@126.com; 4 Department of Pediatrics, Yueyang Maternal and Child Health Hospital, 693 Baling Middle Rd., Yueyang 414000, China; xugaosheng0414@163.com; 5 Department of Obstetrics and Gynecology, the First Affiliated Hospital of Baotou Medical School, 41 Linyin Rd., Baotou 014000, China; byyfygh2010@163.com; 6 Wyeth Nutrition Science Center, 582 Wuzhong Rd., Shanghai 201103, China; Manxi.Bai@wyethnutrition.com

**Keywords:** docosahexaenoic acid, lactating women, food frequency questionnaire, breast milk, plasma, erythrocyte, correlation

## Abstract

We aimed to assess the correlation between docosahexaenoic acid (DHA) dietary intake and the plasma, erythrocyte and breast milk DHA concentrations in lactating women residing in the coastland, lakeland and inland areas of China. A total of 408 healthy lactating women (42 ± 7 days postpartum) were recruited from four hospitals located in Weihai (coastland), Yueyang (lakeland) and Baotou (inland) city. The categories of food containing DHA, the average amount consumed per time and the frequency of consumption in the past month were assessed by a tailored DHA food frequency questionnaire, the DHA Intake Evaluation Tool (DIET). DHA dietary intake (mg/day) was calculated according to the Chinese Food Composition Table (Version 2009). In addition, fasting venous blood (5 mL) and breast milk (10 mL) were collected from lactating women. DHA concentrations in plasma, erythrocyte and breast milk were measured using capillary gas chromatography, and were reported as absolute concentration (μg/mL) and relative concentration (weight percent of total fatty acids, wt. %). Spearman correlation coefficients were used to assess the correlation between intakes of DHA and its concentrations in biological specimens. The study showed that the breast milk, plasma and erythrocyte DHA concentrations were positively correlated with DHA dietary intake; corresponding correlation coefficients were 0.36, 0.36 and 0.24 for relative concentration and 0.33, 0.32, and 0.18 for absolute concentration (*p <* 0.05). The median DHA dietary intake varied significantly across areas (*p <* 0.05), which was highest in the coastland (24.32 mg/day), followed by lakeland (13.69 mg/day), and lowest in the inland (8.84 mg/day). The overall relative and absolute DHA concentrations in breast milk were 0.36% ± 0.23% and 141.49 ± 107.41 μg/mL; the concentrations were significantly lower in inland women than those from coastland and lakeland. We conclude that DHA dietary intake is positively correlated with DHA concentrations in blood and breast milk in Chinese lactating women, suggesting that the tailored DHA food frequency questionnaire, DIET, is a valid tool for the assessment of DHA dietary intake.

## 1. Introduction

Docosahexaenoic acid (DHA, 22:6*n*-3) plays an important role in infant growth and development, especially during early postnatal months, a period of rapid brain growth [1,2]. The accretion of DHA into brain during this period mainly depends on dietary sources [3]. Therefore, for breastfeeding infants, their nutritional status concerning DHA is largely determined by their mothers’ DHA status [4,5]. Many professional organizations, including the Chinese Nutrition Society, recommend lactating women to consume a minimum of 200 mg DHA per day in order to meet their own needs and to achieve optimal growth of infants [6,7]. However, as noted by the European Commission with the International Society for the Study of Fatty Acids and Lipids, only 25% of women at 3 months postpartum met the recommendation [8]. In China, there is a scarcity of relevant data for lactating women [9], possibly because there is no valid tool for the assessment of DHA intake.

The food frequency questionnaire (FFQ) has been suggested as an optimal tool in estimating dietary intake of DHA as it cannot be synthesized *in vivo* and primarily comes from aquatic products [10,11]. Most FFQs available in the literature covered the whole diets and were originally designed to assess a wide range of nutrients, which were quite lengthy and not ideal for dietary assessment focusing specifically on DHA [12,13]. Given that DHA is contained in only a small range of foods, efforts have also been made to develop a tailored DHA specific FFQ [14,15], which demonstrated comparable validity (0.42–0.52) as compared with the whole diet-based FFQs (0.19–0.54) [16]. To our knowledge, previous studies were primarily conducted in the general populations but none in lactating women [16]. Due to the possibility of changed dietary patterns and different metabolic profile of fatty acid during lactation [17], the validity of FFQ in assessing dietary DHA intake of lactating women remains to be determined.

In this study, we aimed to evaluate the performance of a newly-developed DHA specific FFQ by assessing the correlation between dietary DHA intake and its concentration in plasma, erythrocyte and breast milk among Chinese lactating women residing in coastland, lakeland and inland areas of China.

## 2. Materials and Methods

### 2.1. Subjects

In this study, we recruited totally 408 healthy lactating women (42 ± 7 days postpartum) from four hospitals located in Weihai (coastland), Yueyang (lakeland) and Baotou (inland) city between May and July of 2014. The inclusion criteria were: (1) healthy women aged 18–35; (2) currently exclusive breastfeeding or partial breastfeeding (women who feed infants formula other than breast milk); (3) having had a singleton pregnancy; (4) local permanent residents. Women were considered not eligible if they had been diagnosed as having severe heart, liver, kidney or lung diseases, had serious mental illness, were allergic to fish, shrimp, shellfish or other DHA-rich food, or had participated in other research projects in the past 30 days. The Institutional Review Board/Human Subjects Committee at Peking University Health Science Center (IRB00001052-14012; date of approval: 22-04-2014) approved the study protocol, and all participating women signed informed consents.

### 2.2. Data Collection

The survey consisted of two parts. Firstly, we collected information about socio-demographic and maternal characteristics, including age, ethnicity, education level, annual family income per capita, height, breastfeeding patterns, pre-pregnancy and postpartum weight of mothers, as well as neonatal birth weight and gender. Then, we collected dietary information of the previous month by a newly designed electronic version DHA specific FFQ, the DHA Intake Evaluation Tool (DIET), where the pictures of various kinds of food and intake reference diagrams were provided. The DIET was designed to capture all food containing DHA listed in the China Food Composition Table (CFCT, Version 2009) [18], including three categories: seafood (75 kinds such as mackerel, lobster and crab), freshwater food (38 kinds such as carp, shrimp and river crab) and mutton. Totally, there were 12 food frequency options ranging from once per month to three times per day, and 9 food intake options ranging from 25 g to 250 g of edible portion. The survey was self-administered by lactating mothers and took approximately 10 min on average to complete. DHA dietary intake was calculated on the basis of the CFCT (Version 2009) [18].

To ensure the data quality, we trained all project staff intensively, standardized data collection procedures across sites, and designated an investigator at each site to supervise the study and reported the study process weekly.

### 2.3. Sample Collection and Analysis

For each participant, fasting venous blood (5 mL) and breast milk (10 mL) were collected. The detailed procedure for blood collection and processing has been described in our previous publication [19]. In brief, the blood samples were collected into an ethylenediaminetetraacetic acid (EDTA)-containing tube, and placed in the refrigerator at 5 °C for 30 min before centrifuging at 3000 rpm for 10 min to separate plasma and erythrocytes. Breast milk samples were collected into a sterile container in the morning (10 ± 2 a.m.) from the non-feeding breast manually or with a breast pump. In the study, mothers were allowed to breastfeed the baby using one side of the breast in the morning, and if a mother did breastfeed her baby, breast milk was collected from the non-feeding breast. If not, breast milk was collected from either side. It has been suggested by a study [20], that fat concentrations in the non-feeding breast are less likely to be influenced by the breastfeeding behavior using the other breast. Both blood and milk samples were stored at the local hospital at −20 °C for about 10 days before being transported on dry ice frozen to the central laboratory where samples were stored at −80 °C in a freezer.

Total lipids in blood and breast milk samples were extracted and derived following a modified method of Folch *et al*. [20], and were analyzed by capillary gas chromatography. The detailed analysis procedure for total lipids in the blood has been descried previously [21]. The same methods were used for breast milk analysis. Briefly, the internal standard solution with methyl ester (C11:0) was added to each sample, and mixed with boron trifluoride and methanol. The mixture was heated at 115 °C for 20 min and extracted with n-hexane after cooling down to room temperature. The n-hexane containing methyl esters of total lipids was analyzed by Agilent 6890N capillary gas chromatography (Agilent Technologies, Palo Alto, CA, USA) equipped with a capillary column (CP-Sil 88, 50 m, 0.25 mm ID, 0.20 μm film thickness). The fatty acids were separated by a programmed temperature ramping method, and the results were recorded via the Agilent Open LAB software (Agilent Technologies, Santa Clara, CA, USA). Both absolute (μg/mL) and relative (weight percent of total fatty acids, wt. %) concentrations of DHA were reported.

### 2.4. Statistical Analysis

The total dietary intake of DHA in the past month was calculated based on food consumption frequency, the average amount consumed per time, and the DHA content in the food. Of the 408 participants, 3 (1 lakeland, 2 inland) with obviously abnormal dietary intake and 9 (3 coastland, 5 lakeland, 1 inland) who had consumed DHA supplements in the past month were excluded; thus, 396 subjects were included in the analysis. There were no missing data on all socio-demographic and maternal characteristics, except that the information on education level and annual family income per capita was missing for 3% and 6% of subjects.

Data were presented as means ± SDs, median (interquartile range), or percentage (%) as appropriate. The statistical differences between regions in DHA dietary intake and the DHA concentrations in blood and breast milk were examined by one-way analysis of variance (ANOVA) and Kruskal-Wallis tests, followed by Tukey’s HSD and Kruskal-Wallis one-way ANOVA by ranks for multiple comparisons, as appropriate. In addition, we explored whether DHA concentrations in breast milk varied across subgroups defined by maternal ethnicity, age (18.0–24.9, 25.0–29.9, and over 30.0 years), pre-pregnancy BMI (<18.5, 18.5–23.9, and 24.0 kg/m^2^), annual family income per capita, education attainment (middle school or less, high school, and college or above), maternal dietary intake and feeding methods by using multiple linear regression with adjustment for region.

Spearman correlation analysis was used to assess the correlation between DHA dietary intake and its concentrations in biological specimens. To facilitate the description of dietary sources of DHA, we defined a category of food (seafood, freshwater food, and mutton) as the major DHA source for an individual if this food category comprised ≥50% of total DHA intake. We compared the regional differences in major DHA sources by using the Chi-square test. Statistical analyses were performed by using SPSS version 20.0 (Chicago, IL, USA). *P* values were two-sided, and *p <* 0.05 was considered statistically significant.

## 3. Results

### 3.1. Maternal Characteristics

The mean age, height, pre-pregnancy BMI, postpartum BMI of the 396 lactating mothers and mean birth weight of their infants were 27.34 ± 2.97 years old, 162.04 ± 4.95 cm, 21.07 ± 3.31 kg/m^2^, 23.61 ± 3.32 kg/m^2^, 3.39 ± 0.46 kg, respectively. The mean annual family income per capita was 27,507 ± 18,789 Chinese Yuan. In total, 95.7% of the mothers were of Han ethnicity, and 36.8% had high school or above education; approximate 60% of mothers breastfed exclusively. Table 1 shows the characteristics of mothers and the infants by region. The overall tests showed that there were significant regional differences in all the characteristics except mothers’ education, ethnics, feeding methods and infants’ gender. Specifically, lakeland women were relatively younger, shorter in stature, and had lower pre-pregnancy BMI and postpartum BMI.

### 3.2. DHA Dietary Intake

Table 2 shows the DHA dietary intake of lactating women by region. The median intake varied significantly across the three areas (*p <* 0.001), which was highest in the coastland (24.32 mg/day), followed by the lakeland (13.69 mg/day), and lowest in the inland (8.84 mg/day). Totally, 126 women consumed mutton (19 in the coastland and 107 in the inland). The mean DHA dietary intake from mutton was 0.15 mg/day and 6.18 mg/day for women residing in coastland and inland.

The major DHA food sources differed significantly by region (χ^2^ = 153.49, *p <* 0.001) (Table 3). In coastland, seafood was the major food source for 48% of lactating mothers and for another 48% was freshwater food. In inland, the major food source for 40% of the mothers was freshwater food and 35% was mutton. In lakeland, the major food source for four fifths of women was freshwater food.

### 3.3. DHA Concentrations in Breast Milk and Multiple Regression Analyses

The overall mean DHA absolute and relative concentrations in breast milk were 141.49 μg/mL and 0.36%, respectively; the concentration was higher for coastland and lakeland than inland mothers in both absolute and relative DHA concentrations (Table 4). Plasma and erythrocyte DHA concentrations have been reported previously [19].

In multiple linear regression analyses, maternal dietary intake and geographical region were significantly associated with both absolute and relative DHA concentrations. Maternal feeding method was significantly associated with absolute DHA concentrations. As the results showed, none of the characteristics (including maternal ethnic, age, pre-pregnancy BMI, annual family income per capita, education attainment) have an impact on DHA concentrations in breast milk.

### 3.4. Spearman Correlation Analysis

In overall analyses, the DHA dietary intake was significantly correlated with its relative concentrations in the breast milk, plasma and erythrocyte; corresponding spearman correlation coefficients were 0.36, 0.36 and 0.24, respectively (*p <* 0.001) (Table 5); similar but slightly lower correlation coefficients were observed for absolute concentrations in the three biomarkers. In stratified analyses by region, moderate correlations of DHA intake with both relative (0.18–0.45) and absolute (0.15–0.41) concentrations in plasma and breast milk were identified in the three regions, although some did not reach statistical significance. The correlations between DHA intake and erythrocyte DHA (relative: 0.05–0.30; absolute: 0.02–0.18) were relatively lower than those of plasma and breast milk DHA.

## 4. Discussion

In this large cross-sectional study conducted in the three typical urban areas of China, the DHA dietary intake was significantly and positively correlated with plasma, erythrocyte, and breast milk DHA concentrations, suggesting the possibility of using the DIET to assess DHA dietary intake. We also found that the DHA dietary intake of lactating women residing in the coastland, lakeland and inland areas of China was substantially lower than the adequate intake (200 mg/day) recommended by the Chinese Nutrition Society [7].

In our study, the breast milk, plasma and erythrocyte DHA concentrations were positively correlated with DHA dietary intake; corresponding correlation coefficients were 0.36, 0.36 and 0.24 for relative concentration and 0.33, 0.32, and 0.18 for absolute concentration. The magnitude of correlation in our study is slightly lower than that reported by a Canadian study and a Norwegian study, both of which also utilized DHA specific FFQ [14,15]. In the Canadian study, the correlation coefficient between DHA dietary intake and whole blood DHA was 0.42 [15], and in the Norwegian study, the correlation coefficient between DHA dietary intake and erythrocyte DHA was 0.52 [14]. The underlying reasons might be complex. Firstly, such difference may be a reflection of race differences in metabolic profile regarding DHA. Secondly, the relatively lower correlation in our study may suggest that our estimation of dietary intake might be less accurate than in aforementioned studies. If so, the reduced precision may be partially because we did not consider the variations in cooking methods of aquatic products. In China, the aquatic products could be cooked in a variety of ways, and it is unrealistic for us to accurately quantify the impacts of various cooking ways on fatty acids. Thirdly, both the Canadian and the Norwegian study were conducted in general population [14,15], whereas our study focused on lactating mothers whose fatty acid metabolic profile might be quite different from other populations [17]. In any case, the magnitude of our correlation for plasma and erythrocyte DHA was comparable to some other studies utilizing the FFQs covering the whole diet [16]. In addition to the correlation between dietary intake of DHA and its concentrations in plasma and erythrocytes, we also identified clear evidence of a positive correlation for breast milk, which further enhances the validity of our findings, suggesting that the DIET is suitable for the estimation of dietary intake of DHA. In the present study, the overall correlation coefficients were generally lower than those of coastland women, but higher than those of lakeland and inland women, which is in accordance with the variations of dietary DHA intake (*i.e.*, the overall interquartile range of intake was 5.54–32.28, the range for coastland women 8.46–57.49, for lakeland women 6.16–25.47, and for inland women 2.80–21.96), suggesting that it is quite important for future studies to validate FFQs in a population with diverse dietary patterns. Moreover, the correlation coefficients for plasma and breast milk were generally higher than those for erythrocytes, which corresponds to the fact that erythrocyte DHA is an indicator of longer term nutritional status [22], whereas plasma and breast milk DHA often reflect short term dietary intake [12,22,23].

In our study, the median DHA dietary intake was highest in lactating mothers residing in the coastland area (24.3 mg/day) and lowest in those from the inland area (8.8 mg/day), and the intake of lakeland mothers (13.7 mg/day) was intermediate between them. Compared to other studies carried out among Chinese population, the dietary intake of our population was relatively lower. On the basis of the whole diet FFQ, three cross-sectional studies reported the dietary intake of DHA of 54.7–93.9 mg/day, 35.4–51.7 mg/day, and 11.8–41.1 mg/day, in coastland, lakeland, and inland pregnant women of China, respectively [24,25,26]. In addition to the differences in study population, our study was carried out during the fish close season when the supply of aquatic products might reduce materially. Another reason might be due to the differences of the questionnaire [27]. Specifically, the food list of DIET was determined strictly following the CFCT (Version 2009) [18], and therefore only food containing DHA indicated by CFCT was included, enabling us to calculate the dietary intake of DHA based on standardized DHA content value for a specific food. However, foods like eggs which have been demonstrated by other studies as a non-negligible food source for DHA were not included in DIET as the CFCT states that DHA in eggs is not detectable [28].

It is noteworthy that the DHA dietary intake in our study as well as other studies conducted in China are much lower than reports from other countries. In developed countries like Spain, Japan and Korea, the dietary intake of the general population based on the whole diet FFQ was 400 mg/day, 290 mg/day and 174 mg/day, respectively [29,30,31]. In the USA, Australia, and Canada, the dietary intake of general population based on DHA specific FFQ were 206 mg/day, 201 mg/day, and 128 mg/day, respectively [21,27,32]; notably, only marine food was included in the FFQ of the USA study. Apparently, even in the coastland of China the DHA dietary intake was only one-fifth of that in Canada and one-tenth of that in USA [21,32]. The DHA intake differences between Chinese and other populations are rather consistent with the differences in aquatic food consumption patterns. Firstly, the intake of aquatic products per capita is substantially lower in China than other countries [33]. For example, the amount of aquatic products consumed by lactating women in China was only 7% of that in Sweden [34]. Secondly, most common seafood consumed in China were scallops and lean fish which contain less fatty acids, while people in other countries usually consumed fatty fish such as salmon and herring with higher content of DHA [26,35]. The Chinese Nutrition Society recommends the adequate intake of DHA as 200 mg/day for lactating women [7]. Obviously, even for women residing in the coastal area, their DHA dietary intake was only one eighth of the recommended value, indicating the necessity for lactating women to consume more fatty fish or take DHA supplements under the guidance of doctors [9].

Interestingly, despite substantially insufficient dietary intake relative to the recommendations, the mean breast milk DHA concentration in our study (0.36%) was slightly higher than the mean level of 65 studies worldwide (0.32%) [36], which is worth studying in the future. Except dietary intake and geographical location, we observed in multiple regression analyses that none of the common characteristics (including maternal ethnic, age, pre-pregnancy BMI, annual family income per capita, education attainment, and feeding methods) seemed to have an important impact on DHA concentrations in breast milk.

Our study has strengths. Firstly, we developed a user-friendly electronic version FFQ where food pictures and intake reference diagrams were provided, which could help the participants to recall more accurately. Secondly, our study was conducted in three areas of China, representing three typical areas with different dietary habits related to aquatic products. Thirdly, compared to other studies in the literature, our study had large sample size and simultaneously assessed the correlation between DHA dietary intake and its concentrations in plasma, erythrocytes, and breast milk.

Our study also has limitations. First, we did not include food that may contain DHA but was not listed in the CFCT (Version 2009). Second, this study is conducted in urban areas, which may limit the generalization of its findings to a broader population because the availability and consumption patterns of aquatic products between rural and urban China may be different. Third, we did not assay α-linolenic acid that can convert to DHA and therefore may be of importance with respect to the interpretation of the correlation between dietary intake of DHA and its concentrations. Additionally, erythrocyte DHA measurements may be lower than the true values because of the temporary storage of blood samples under −20 °C [37,38], and consequently, the correlation between dietary intake DHA and its concentrations in erythrocytes may be slightly compromised.

## 5. Conclusions

To summarize, this study is the first in China to assess the correlation between DHA dietary intake and its concentrations of biological specimens in lactating women. The DHA dietary intake is positively correlated with the three biomarkers, suggesting that the tailored FFQ can be used to assess the dietary intake of DHA. Consistent with the findings from other studies, the DHA dietary intake of lactating women in China is substantially inadequate. Given the importance of DHA nutritional status of lactating women on the health of both mothers and infants, it is of great significance to improve the maternal DHA nutritional status.

## Figures and Tables

**Table 1:** Characteristics of the mothers and infants by region.

Characteristics	Total (*n* = 396)	Coastland (*n* = 133)	Lakeland (*n* = 130)	Inland (*n* = 133)	*p* Value
**Mothers:**					
Age (year)	27.34 ± 2.97	27.79 ± 2.69 ^a^	26.61 ± 3.08 ^b^	27.60 ± 3.00 ^a^	<0.01
Ethnics (%)					>0.05
Han	95.7	97.0	97.7	92.5	
Others	4.3	3.0	2.3	7.5	
Education (%)					>0.05
College or above	36.8	38.6	32.5	39.2	
High school	52.5	51.5	53.2	52.8	
Middle school or less	10.7	9.9	14.3	8.0	
Annual family income per capita	27,507 ± 18,789	21,474 ± 11,291 ^a^	30,298 ± 23,201 ^b^	31,579 ± 18,524 ^b^	<0.001
Height (cm)	162.04 ± 4.95	163.28 ± 5.05 ^a^	159.75 ± 4.31 ^b^	163.05 ± 4.68 ^a^	<0.001
Pre-pregnancy BMI (kg/m^2^)	21.07 ± 3.31	21.88 ± 3.52 ^a^	20.12 ± 2.96 ^b^	21.19 ± 3.22 ^a^	<0.001
Postpartum BMI (kg/m^2^)	23.61 ± 3.32	24.52 ± 3.52 ^a^	22.54 ± 2.92 ^b^	23.75 ± 3.20 ^a^	<0.001
Feeding methods (%)					>0.05
Exclusively breastfeeding	58.8	54.1	63.8	58.6	
Partially breastfeeding	41.2	45.9	36.2	41.4	
**Infants:**					
Birth weight (kg)	3.39 ± 0.46	3.46 ± 0.43 ^a^	3.28 ± 0.44 ^b^	3.42 ± 0.48 ^a^	<0.01
Gender, Male (%)	53.5	51.9	46.9	61.7	>0.05

Means ± SDs within a row with unlike superscript letters are significantly different (*p <* 0.05).

**Table 2:** Docosahexaenoic acid (DHA) dietary intake of lactating women by region (mg/day).

Dietary Sources of Foods	All (*n* = 396)	Coastland (*n* = 133)	Lakeland (*n* = 130)	Inland (*n* = 133)	*p* Value
**Seafood**					
Mean ± SDs	18.37 ± 33.23	27.89 ± 39.73 ^a^	9.98 ± 25.07 ^b^	13.43 ± 26.40 ^b^	<0.001
Median (interquartile range)	3.28 (0.48, 20.72)	12.26 (1.65, 31.42) ^a^	1.70 (0, 7.81) ^b^	1.20 (0, 10.01) ^b^	<0.001
**Freshwater food**					
Mean ± SDs	14.75 ± 24.42	19.76 ± 32.08 ^a^	17.12 ± 22.50 ^a^	7.56 ± 14.07 ^b^	<0.001
Median (interquartile range)	5.97 (2.18, 15.63)	7.44 (2.79, 19.54) ^a^	10.63 (4.42, 21.33) ^a^	2.95 (0.76, 7.46) ^b^	<0.001
**Mutton**					
Mean ± SDs	2.13 ± 8.55	0.15 ± 0.87 ^a^	0 ± 0 ^a^	6.18 ± 13.90 ^b^	<0.001
Median (interquartile range)	0 (0, 0.43)	0 (0, 0) ^a^	0 (0, 0) ^a^	1.84 (0.22, 5.53) ^b^	<0.001
**Total food intake**					
Mean ± SDs	30.00 ± 43.85	44.37 ± 56.77 ^a^	24.20 ± 32.95 ^b^	21.29 ± 33.95 ^b^	<0.001
Median (interquartile range)	13.42 (5.54, 32.28)	24.32 (8.46, 57.49) ^a^	13.69 (6.16, 25.47) ^b^	8.84 (2.80, 21.96) ^c^	<0.001

Means ± SDs or median (interquartile range) within a row with unlike superscript letters are significantly different (*p <* 0.05).

**Table 3:** Major food source ^a^ of DHA by region.

The Major DHA Source	Coastland		Lakeland		Inland	
	*n*	%	*n*	%	*n*	%
Seafood	64	48.12	23	17.69	22	16.54
Freshwater food	64	48.12	107	82.31	53	39.85
Mutton	2	1.50	-	-	47	35.34
None	3	2.26	-	-	11	8.27

^a^ If a category of food (seafood, freshwater food, or mutton) contributed to ≥50% of total DHA intake for an individual, this category of food was defined as the major food source of DHA for this individual. If all the three categories of food contributed to <50% of total DHA intake for an individual, we considered that there was no specific major food source of DHA for this individual.

**Table 4:** DHA concentrations in breast milk and multiple linear regression.

Variables	Breast Milk DHA (μg/mL)				Breast Milk DHA (wt. %)			
	Mean ± SDs	β	95% CI	*p*	Mean ± SDs	β	95% CI	*p*
**Region**								
Inland	113.09 ± 72.90	Ref.	Ref.	-	0.28 ± 0.19	Ref.	Ref.	-
Coastland	163.36 ± 133.82	29.69	8.61–48.01	<0.01	0.43 ± 0.28	0.09	0.05–0.13	<0.01
Lakeland	148.17 ± 100.98	−2.34	−27.85–20.95	0.85	0.38 ± 0.17	−0.03	−0.08–0.02	0.26
**Age (year)**								
<25.0	150.06 ± 121.28	Ref.	Ref.	-	0.36 ± 0.19	Ref.	Ref.	-
25.0–29.9	135.12 ± 90.72	−33.08	−66.81–1.96	0.06	0.36 ± 0.24	−0.02	−0.08–0.04	0.54
≥30.0	161.98 ± 152.89	−16.75	−58.14–28.83	0.45	0.27 ± 0.19	−0.04	−0.11–0.04	0.34
**Ethnicity**								
Others	128.29 ± 117.84	Ref.	Ref.	-	0.38 ± 0.44	Ref.	Ref.	-
Han	142.08 ± 107.05	−3.50	−85.34–48.13	0.91	0.36 ± 0.21	−0.06	−0.33–0.10	0.64
**Education**								
Middle school or less	124.00 ± 92.11	Ref.	Ref.	-	0.34 ± 0.18	Ref.	Ref.	-
High school	138.18 ± 103.90	16.52	−12.85–44.42	0.27	0.36 ± 0.23	0.002	−0.05–0.05	0.94
College or above	153.61 ± 117.84	25.26	−5.58–56.55	0.10	0.38 ± 0.24	0.005	−0.048–0.06	0.86
**Pre-pregnancy BMI**								
<18.5	139.93 ± 95.16	Ref.	Ref.	-	0.38 ± 0.27	Ref.	Ref.	-
18.5–23.9	143.25 ± 111.83	9.29	−15.89–34.13	0.48	0.36 ± 0.20	−0.02	−0.08–0.04	0.62
≥24.0	136.51 ± 105.04	−2.41	−34.60–32.78	0.86	0.25 ± 0.27	−0.04	−0.14–0.04	0.34
**Annual family income per capita (ten thousand Yuan)**		2.66	−4.59–9.70	0.50		0.01	−0.01–0.02	0.58
**Feeding method**								
Exclusive breastfeeding	131.20 ± 99.74	Ref.	Ref.	-	0.35 ± 0.24	Ref.	Ref.	-
Partial breastfeeding	156.20 ± 116.26	24.68	4.65–43.48	<0.05	0.38 ± 0.21	0.03	−0.01–0.07	0.12
**Dietary DHA intake**		0.75	0.44–1.15	<0.01		0.002	0.001–0.003	<0.01

β, regression coefficient; Ref., reference category.

**Table 5:** Spearman correlation analysis between dietary DHA intake and DHA levels in biomarkers.

Biomarkers	All		Coastland (*n* = 133)		Lakeland (*n* = 130)		Inland (*n* = 133)	
	*r* (95%CI)	*p*	*r* (95%CI)	*p*	*r* (95%CI)	*p*	*r* (95%CI)	*p*
**wt. %**								
Breast milk	0.36 (0.26–0.45)	<0.001	0.45 (0.30–0.58)	<0.001	0.16 (−0.02–0.33)	0.06	0.23 (0.05–0.38)	<0.01
Plasma	0.36 (0.27–0.45)	<0.001	0.46 (0.31–0.60)	<0.001	0.21 (0.03–0.38)	<0.05	0.18 (0.01–0.34)	<0.05
Erythrocyte	0.24 (0.14–0.33)	<0.001	0.30 (0.14–0.44)	<0.01	0.05 (−0.13–0.23)	0.55	0.08 (−0.10–0.25)	0.38
**μg/mL**								
Breast milk	0.33 (0.23–0.42)	<0.001	0.39 (0.23–0.54)	<0.001	0.23 (0.06–0.41)	<0.01	0.25 (0.07–0.42)	<0.01
Plasma	0.32 (0.23–0.41)	<0.001	0.41 (0.26–0.55)	<0.001	0.21 (0.04–0.39)	<0.05	0.15 (−0.01–0.32)	0.08
Erythrocyte	0.18 (0.08–0.27)	<0.001	0.17 (0.01–0.32)	0.06	0.18 (−0.01–0.35)	0.05	0.02 (−0.16–0.20)	0.82
